# Oligodendroglioma Arising in Mature Cystic Teratoma

**DOI:** 10.1155/2014/745462

**Published:** 2014-03-16

**Authors:** Betül Ünal, Faruk Güleç, Murat Şedele

**Affiliations:** ^1^Department of Pathology, Medical Faculty, Akdeniz University, Antalya, Turkey; ^2^Department of Pathology, Antalya Training and Research Hospital, Antalya, Turkey

## Abstract

*Background.* Development of neuroepithelial tumors from mature cystic teratoma is very rare. We present a case of oligodendroglioma developing inside mature cystic teratoma. *Case.* Eighteen-years-old female, right adnexal mass with solid and cystic areas was detected. Sections showed all three germ layers. Also, a tumoral lesion was observed in a glial fibrillary matrix. Tumor was composed of monotonous, uniform cells which have oval-round nucleus, perinuclear halo, and indistinct cytoplasm. GFAP, EGFR, P53 were positive. *Conclusions.* We diagnosed oligodendroglioma arising from mature cystic teratoma. There was no recurrence at the end of 13 months followup. The number of cases which have been reported in the literature is only a few.

## 1. Introduction

Mature cystic teratoma is the most common neoplasia of the ovary and it originates from all three germ layers (endoderm, mesoderm, and ektoderm). Ovarian teratomas account for 25% of all ovarian tumors [1]. Malignancies can develop from teratomas in the elderly, especially after the fifth decade. The most common malignancy originating from mature cystic teratoma is squamous cell carcinoma [2]. Additionally, adenocarcinoma, undifferentiated carcinoma, sarcoma, papillary carcinoma, and malignant melanoma can also develop [3]. But the development of neuroepithelial tumors is very rare. We present a case of oligodendroglioma developing inside mature cystic teratoma.

## 2. Case Report

An eighteen-year-old female was admitted to our hospital with abdominal pain. On physical examination, on the right side of the abdomen a palpable mass was detected. Ultrasonography showed right adnexal mass with solid and cystic areas inside. After laparotomy and oophorectomy, pathological examination was performed.

### 2.1. Gross Evaluation

Oophorectomy material had smooth surface and consisted of solid and cystic areas. On the cut surface mature adipose tissue, bone, cartilage, and hair and, in an area of about 6 cm soft, gray-pink, solid-microcystic lesion were observed.

### 2.2. Microscopic Evaluation

**Figure 1:**
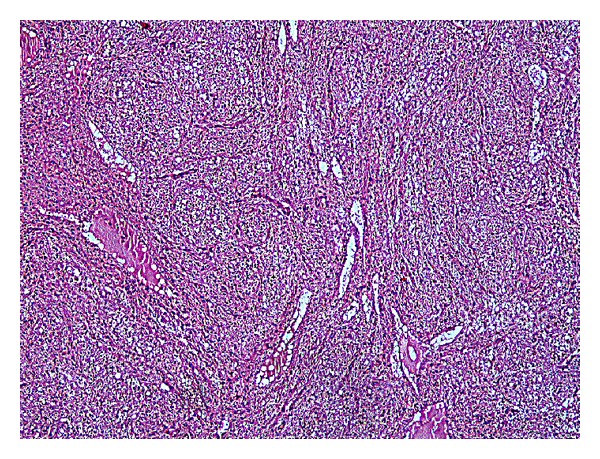
Tumoral lesion inside mature cystic teratoma with glial fibrillary matrix.

**Figure 2:**
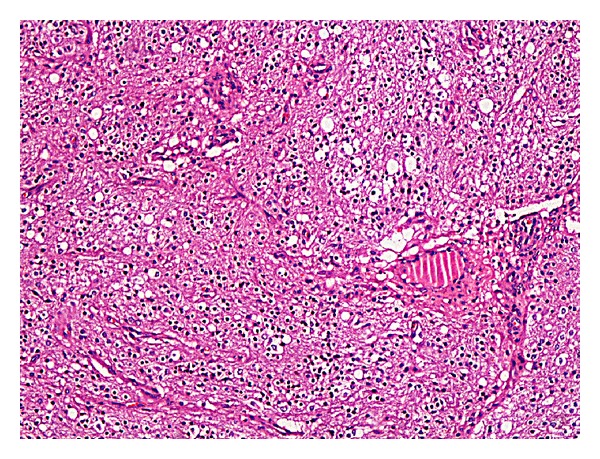
The sections showing a monotonous population of round, uniform cells with a hyperchromatic nucleus and perinuclear halo.

In many sections tissues belonging to all three germ layers (adipose tissue, cartilage, bone, choroid plexus, nerve tissue, mucinous epithelium, etc.) were seen (Figures 1 and 2).

**Figure 3:**
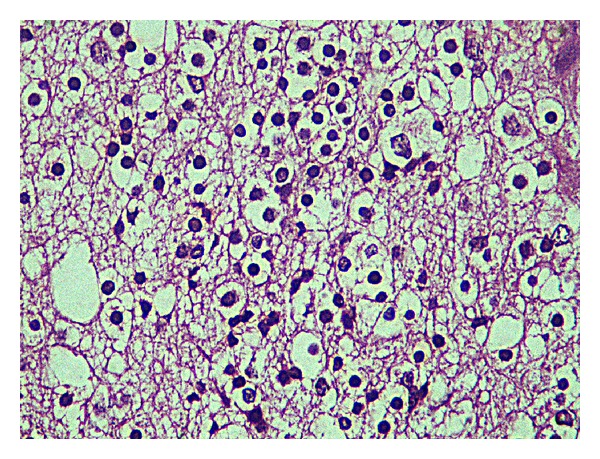
“Fried-egg” appearance of oligodendroglioma.

Also, an area of about 6 cm tumoral lesion was observed in a glial fibrillary matrix. Tumor was composed of monotonous, uniform cells which have oval-round nucleus, perinuclear halo, and indistinct cytoplasm (Figures 1, 2, and 3).

### 2.3. Immunohistochemical Analysis

**Figure 4:**
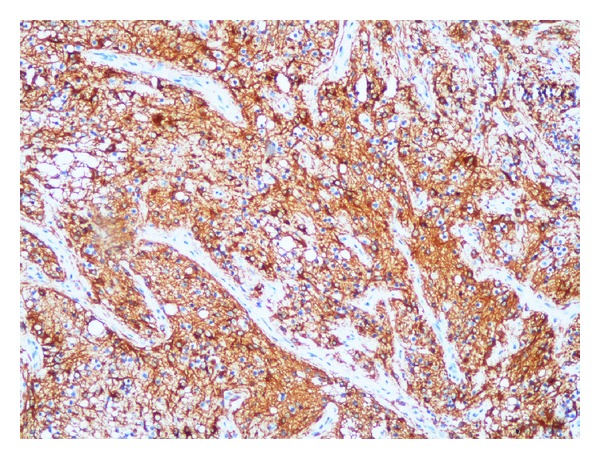
GFAP positivity in tumor cells.

In this tumoral area, GFAP (Figure 4), EGFR, and P53 were positive and Ki-67 proliferating index was 2–5%. In the present findings, we diagnosed oligodendroglioma arising in mature cystic teratoma.

## 3. Discussion

Squamous cell carcinomas account for 80% of the malignancies arising in mature cystic teratomas [2]. Apart from this, tumors originating from neural tissue are rare, and the cooccurrence of central neurocytomas, ependymomas, glioblastomas, neuroblastomas, neuroectodermal tumors, and multiple neuroectodermal tumors together with mature cystic teratomas has been reported in the literature [4–7]. Oligodendrogliomas arising in teratomas are extremely rare, and, to our knowledge, three cases in the adulthood [3, 8, 9] and one case in the childhood [10] have been reported in the literature. Furthermore, Din et al. reviewed six cases and reported that oligodendrogliomas can arise in mature and immature teratomas and those arising in immature teratomas have the worst prognosis [11].

Oligodendrogliomas are malignant tumors originating from oligodendrocytes and arising in the cerebral hemispheres in the young and middle ages. The macroscopic examination shows a soft, shiny, gray-pink colored, solid mass with focal cystic and calcific degenerations. The microscopic examination shows a monotonous population of round, uniform cells with a hyperchromatic nucleus and perinuclear halo. The perinuclear halo observed with H&E staining is a fixation artifact and represents a fried-egg appearance typical of oligodendrogliomas. In addition, anastomosis of fine capillary network is termed as a “chicken wire” appearance. Calcification is more frequent in such tumors.

Immunohistochemistry has a limited place in the diagnosis of oligodendrogliomas, and the diagnosis is essentially based on the histomorphological findings and the exclusion of other diagnoses. GFAP, NF protein, S100, and Leu-7 can be useful in the diagnostic process. Dot-like EMA positivity and GFAP positivity particularly in the perivascular zone observed in ependymomas are helpful markers in differentiating oligodendrogliomas from clear cell ependymomas. In addition, synaptophysin immunoreactivity is a typical finding for central neurocytomas, which are also clear cell-type tumors.

Surgical resection is a curative treatment in mature cystic teratomas. In the reported cases until today, surgery achieved up to four years of disease-free survival in patients with oligodendrogliomas arising in the teratoma. The current case remained recurrence-free at the end of 13 months of followup. However, long-term follow-up results are required to structure a treatment approach. Furthermore, the possibility of various malignancies in the teratoma should be kept in mind, and multiple samplings should be performed from different sites.

## References

[1] Mills ES, Carter D, Greenson JK (2004). *Sternberg’s Diagnostic Surgical Pathology*.

[2] Thaker S (2012). Squamous cell carcinoma developing in mature cystic teratoma of the ovary: a rare case. *Journal of Obstetrics & Gynaecology*.

[3] Zannoni GF, Fadda G, Scambia G, Capelli A, Carbone A (2002). Oligodendroglioma arising within a mature cystic ovarian teratoma: case report and review of the literature. *Acta Obstetricia et Gynecologica Scandinavica*.

[4] Hirschowitz L, Ansari A, Cahill DJ, Bamford DS, Love S (1997). Central neurocytoma arising within a mature cystic teratoma of the ovary. *International Journal of Gynecological Pathology*.

[5] Oláh KS, Needham PG, Jones B (1989). Multiple neuroectodermal tumors arising in a mature cystic teratoma of the ovary. *Gynecologic Oncology*.

[6] Yadav A, Lellouch-Tubiana A, Fournet JC (1999). Glioblastoma multiforme in a mature ovarian teratoma with recurring brain tumours. *Histopathology*.

[7] Unal E, Koksal Y, Toy H, Gunel E, Acikgozoglu S (2010). Neuroblastoma arising from an unresected sacrococcygeal teratoma in a child. *Journal of Pediatric Hematology/Oncology*.

[8] Opris I, Ducrotoy V, Bossut J, Lamy A, Sabourin J-C (2009). Oligodendroglioma arising in an ovarian mature cystic teratoma. *International Journal of Gynecological Pathology*.

[9] Caltabiano R, Lanzafame S (2008). Oligodendroglioma arising in an immature ovarian teratoma: case report. *Pathologica*.

[10] Bay SB, Corapcioglu F, Kavurt S, Müezzinoğlu B, Anik Y, Tugay M (2010). Oligodendroglioma arising in a mature cystic ovarian teratoma in a child. *Pediatric Hematology and Oncology*.

[11] Din NU, Memon A, Aftab K, Ahmad Z, Ahmed R, Hassan S (2012). Oligodendroglioma arising in the glial component of ovarian teratomas: a series of six cases and review of literature. *Journal of Clinical Pathology*.

